# Human-Governed Validation of Artificial Intelligence-Generated Medical Assessment Artifacts: A Technical Report

**DOI:** 10.7759/cureus.115344

**Published:** 2026-08-28

**Authors:** Vinicius C Destefani, Matheus Feliciano C Ferreira, Afranio C Destefani

**Affiliations:** 1 Medical Education, Centro Universitário Integrado, Campo Mourão, BRA; 2 School of Medicine, Escola Superior de Ciências da Santa Casa de Misericórdia de Vitória, Vitória, BRA

**Keywords:** educational measurement, generative ai, medical education, multiple-choice questions, psychometrics, quality assurance

## Abstract

Generative artificial intelligence (AI) models can create fluent medical assessment materials, including multiple-choice questions, distractors, clinical vignettes, answer explanations, and blueprint tags. However, linguistic plausibility does not establish measurement validity. Using AI-generated text as ready-made assessment material may introduce risks related to clinical accuracy, item-writing quality, blueprint alignment, distractor functioning, fairness, and score interpretation. This technical report presents a human-governed validation workflow that treats AI-generated medical assessment artifacts as candidates requiring staged review rather than immediate deployment. The workflow is organized into seven gates: artifact taxonomy, AI provenance and human accountability, clinical and content review, item-writing and language review, blueprint alignment, psychometric-readiness decision, and final human approval. The psychometric-readiness gate explicitly asks whether item analysis, distractor analysis, differential item functioning (DIF) review, or other empirical evaluation is required before an artifact is used in a scored assessment. By separating expert clinical review from psychometric evidence, the workflow preserves human accountability while acknowledging that expert approval alone cannot support all score-based interpretations. We recommend introducing AI-generated assessment materials into medical education pipelines as preliminary candidates subject to documented governance, human oversight, and empirical evaluation when used for scored or consequential assessment.

## Introduction

Medical education teams increasingly encounter artificial intelligence (AI)-generated assessment artifacts, ranging from multiple-choice questions (MCQs) and distractors to answer explanations, feedback prompts, and curriculum blueprint tags [1]. While generative models can produce fluent and contextually plausible medical text, the central risk in assessment is treating that fluency as evidence of validity [2,3]. Plausibility does not establish score interpretation, and unverified AI output may introduce clinical inaccuracies, flawed item structures, weak distractors, blueprint mismatch, or construct-irrelevant variance into examinations.

Recent literature on AI-generated MCQs in medical education supports a cautious interpretation of their assessment role. Reviews and comparative studies describe rapid generation, expert ratings, item difficulty, discrimination indices, and other forms of validity evidence, but they also emphasize that current evidence does not justify unsupervised use in summative or consequential assessment [1,4,5]. Practical guidance similarly frames generative AI as a tool for supporting item writers rather than replacing expert review, quality control, and psychometric evaluation [6].

To mitigate these risks, validation should separate several questions that are often conflated. Human clinical review, item-writing quality checks, blueprint alignment, distractor functioning, fairness review, and psychometric evidence answer different questions [7-10]. A workflow that merges these steps may prematurely approve an item that is clinically accurate but psychometrically weak or reject a useful draft artifact because it has not yet been assigned the correct evidentiary burden.

This technical report describes a structured validation workflow for AI-generated medical assessment artifacts, derived from practice as described below. It establishes a formal separation between artifact generation, human review, and readiness for psychometric testing. This report does not evaluate educational effectiveness, student learning, platform performance, item equivalence, or psychometric outcomes. Instead, it details procedural gates intended to preserve editorial integrity and human accountability when AI-assisted tools are used in assessment development [11-13].

The workflow described here was not derived a priori. It emerged from routine practice in an item-development operation producing calibrated formative assessments for Brazilian undergraduate medical programmes, where AI-assisted drafting was introduced into an existing item-writing pipeline. The reviewers involved combined clinical practice and medical education, institutional assessment management, and psychometrics. Rather than adopting a single external standard, we consolidated requirements already established in the literature: item-writing guidelines for structural review [3,8,14], blueprint and construct-alignment principles [2,7], psychometric criteria for item analysis, distractor functioning and differential item functioning (DIF) [9,10], and the International Committee of Medical Journal Editors (ICMJE) and Committee on Publication Ethics (COPE) positions on authorship and accountability for AI-assisted work [11-13]. The gate structure was refined over successive cycles of item development during 2025-2026, as artifacts that passed one form of review continued to fail another, most often items that were clinically sound but structurally flawed, which is what led us to separate clinical review from item-writing review. We did not use a formal consensus methodology such as a Delphi process, and we have not evaluated the workflow empirically. We describe its provenance so that readers can judge its transferability, rather than assume a more formal development process than the one that took place.

## Technical report

Seven gates comprising the proposed workflow

**Figure 1:**
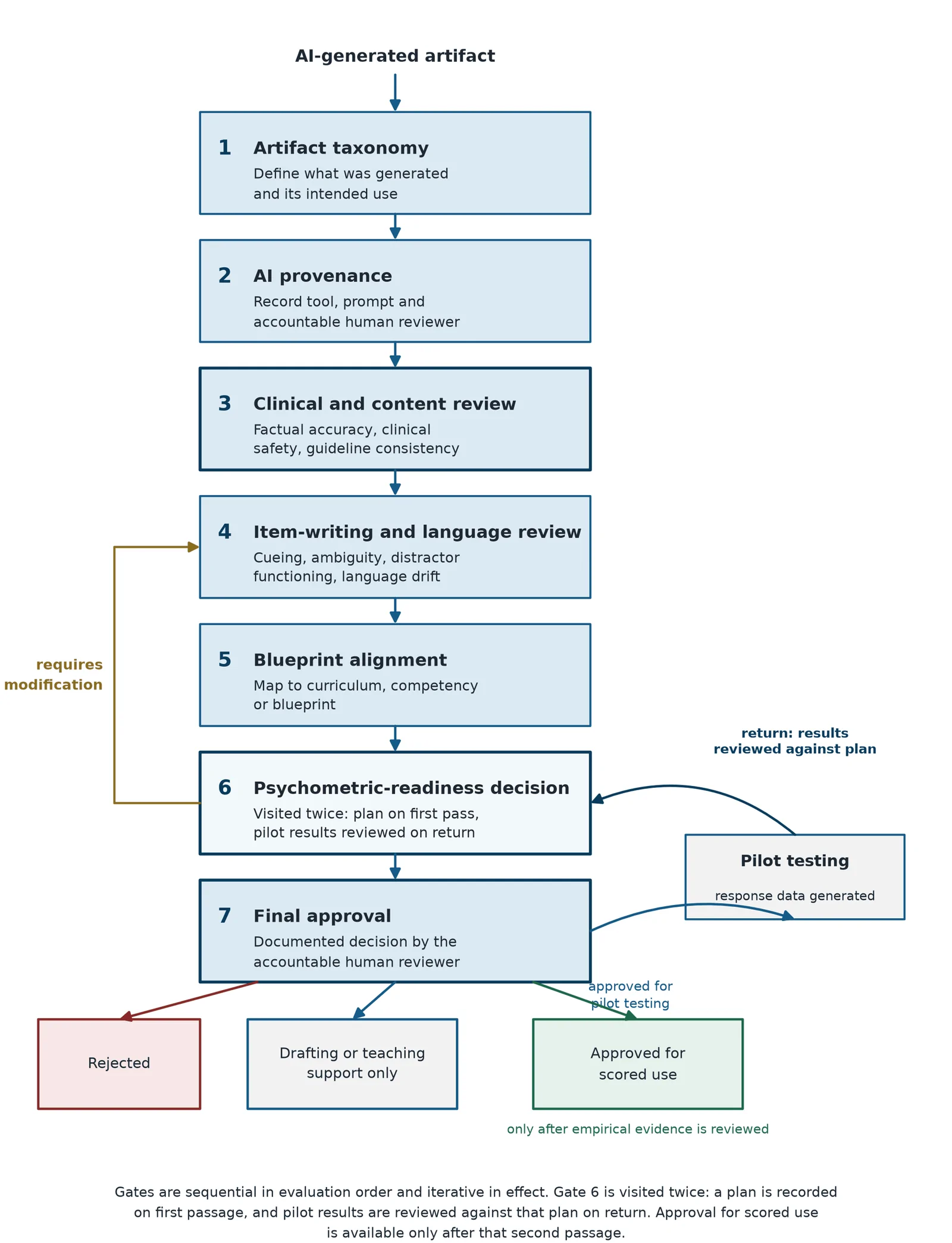
Human-governed validation workflow for AI-generated medical assessment artifacts. Gates are traversed in evaluation order but are iterative in effect. Artifacts classified as requiring modification return to item-writing review. Items approved for pilot testing generate response data and return to the psychometric-readiness gate, where results are reviewed against the documented plan; approval for scored use is available only after that review. AI: artificial intelligence

The proposed validation workflow comprises seven gates. Each gate requires a specific evaluation and a documented human decision, designed to prevent artifacts from bypassing necessary quality controls. The gates are sequential in the order in which they are evaluated, but iterative in effect: an artifact that fails a gate, or that is found deficient once empirical data become available, returns to the appropriate earlier gate rather than being abandoned. Table 1 summarizes the gates and their stop conditions, and Figure 1 shows the sequence together with the revision loops, the rejection path, and the pilot-testing branch.

**Table 1:** AI assessment artifact validation gates. AI: artificial intelligence

Gate	Purpose	Evidence artifact	Stop condition
Artifact taxonomy	Define what was generated and its intended use	Artifact registry; taxonomy field	Artifact family or use case unclear
AI provenance	Preserve transparency and accountability	AI-use log; human reviewer attestation	Generation source or human owner unclear
Clinical/content review	Ensure factual safety and guideline consistency	Clinical subject matter expert sign-off	Unsafe, inaccurate, or outdated content
Item-writing review	Detect cueing, ambiguity, flawed or non-functioning distractors, and language drift	Item-writing checklist; distractor review when data exist	Construct-irrelevant variance or non-functioning options identified; artifact returns for modification or is rejected
Blueprint alignment	Map artifact to a specific learning domain	Curriculum map or item tag	Mismatch with intended competency
Psychometric readiness	Document plan for empirical evidence on first passage; review pilot results against that plan on return	Test plan; pilot analysis plan; reviewed pilot results	Item intended for scored use without psychometric plan or with pilot results not yet reviewed
Final approval	Record deployment status and crystallize human accountability	Final approval flag with outcome category	Missing any prior required gate

Artifact Taxonomy Gate

Before an artifact can be evaluated, its intended function must be defined. AI-generated objects in medical education are not homogeneous; they may include complete MCQs, isolated distractors, clinical vignettes, answer explanations, blueprint tags, feedback prompts, or tutoring prompts. Different artifact families carry different validation burdens [2]. A generated feedback prompt may require clinical safety review but not classical test theory (CTT) calibration, whereas a generated MCQ intended for summative assessment requires stronger evidence. Categorizing the artifact helps prevent the misapplication of validation standards.

AI Provenance and Human Accountability

To preserve transparency, the workflow must record the AI provenance of the artifact, including the tool used, the nature of the prompt, and the human reviewer responsible for the final decision. AI tools cannot be authors and cannot assume responsibility for assessment use [11-13]. Therefore, an accountable human reviewer must be explicitly linked to the artifact from generation to final approval.

Clinical and Content Review

A qualified subject matter expert must review the artifact for factual accuracy, clinical safety, guideline consistency, and unacceptable omissions. This gate serves as a hard stop for unsafe or clinically incorrect material. However, clinical accuracy does not establish measurement quality; an item can be medically accurate yet fail to measure the intended construct or distinguish examinees appropriately [7,8].

Item-Writing and Language Review

Artifacts intended as test items must undergo structural review against established item-writing guidelines [3,8,14]. Reviewers should check for cueing, ambiguity, implausible or non-functioning distractors, option length clues, grammatical cues, and language features that introduce construct-irrelevant difficulty. When response data are available, distractor analysis can identify options that are rarely selected or otherwise fail to function as plausible alternatives [9]. Newly constructed items have no such data by definition; for these, structural review relies on the guidelines alone, and empirical scrutiny is deferred to the psychometric-readiness gate. Artifacts that fail this gate are classified as requiring modification and return to item writing for revision or are rejected outright when the defect cannot be repaired.

Blueprint Alignment

An assessment artifact is only useful if it measures an intended construct. In this gate, the artifact is mapped to a curriculum, competency, domain, or blueprint [2,7]. If an AI-generated item cannot be defensibly mapped to the intended exam blueprint, it should not be used as assessment material, regardless of its clinical accuracy or surface fluency.

Psychometric-Readiness Decision

Expert enthusiasm is not a substitute for empirical evidence [7,8]. If an artifact is intended for scored assessment, the review team must explicitly decide whether pilot testing, item difficulty and discrimination analysis, distractor analysis, DIF/fairness review, or another psychometric process is required before operational use [9,10]. DIF review is particularly relevant when assessment scores may affect comparisons across learner groups, because apparently similar items may function differently across groups after controlling for the measured construct [10]. Non-scored drafting artifacts may proceed with a lighter evidence burden, but any item intended to affect scoring, progression, or certification requires a documented plan for empirical evaluation.

This gate is visited twice for any item that requires empirical evidence. On first passage, it produces a plan, since a newly written item has no response data. Once the item has been piloted, it returns here so that the resulting data are reviewed against that plan: difficulty and discrimination indices, distractor functioning, and DIF where group comparisons are at stake. An item whose pilot data are unsatisfactory is classified as requiring modification and returns to item-writing review or is rejected. Only an item whose empirical results have been reviewed and accepted at this second passage may proceed to approval for scored use.

Final Approval and Limitations

The final gate requires an explicit, documented decision by the accountable human reviewer. The artifact may be classified as rejected; as requiring modification, when a defect in content or structure is minor enough to be repaired and the artifact returns to the appropriate earlier gate; as approved for drafting or teaching support only; as approved for pilot testing, which entails return to the psychometric-readiness gate once data exist; or as approved for scored use, which is available only after empirical evidence has been reviewed. This final sign-off crystallizes human accountability and prevents AI-generated content from being deployed autonomously.

Outcome categories at final approval are as follows: rejected; requires modification; approved for drafting or teaching support only; approved for pilot testing; and approved for scored use.

As summarized in Table 1, these gates provide a structured control mechanism for AI-generated assessment artifacts.

Illustrative decision criteria for the psychometric-readiness gate

The psychometric-readiness gate is specified as a plan-then-review gate rather than as a fixed threshold, because the numerical criteria that make an item acceptable are not portable across measurement frameworks. Each metric below is stated as a chain: definition, prespecified criterion, observed result, and the action that follows when the criterion is not met. The values named are illustrative of criteria used in published work, not values this framework imposes.

Within CTT, item difficulty is the proportion of examinees answering correctly, $\mathrm{p}=\mathrm{c/n}$, where c is the number of correct responses and n is the number of examinees reaching the item. Higher p denotes an easier item. The framework prescribes no universal acceptable range, because the defensible range depends on the decision the scores support: an item that is uninformative for norm-referenced ranking may be appropriate near a criterion-referenced cut score, where items are wanted at the difficulty of the decision. The gate requires that the range be fixed in advance with its rationale and that items outside it be recorded rather than silently retained.

Item discrimination is expressed as the correlation between the item score and the total score. The gate requires the corrected, or item-rest, point-biserial correlation, in which the item is excluded from the total with which it is correlated; including the item introduces a part-whole dependency that inflates the coefficient. With M₁ and M₀ being the mean rest-scores of examinees answering correctly and incorrectly, S the standard deviation of rest-scores, and p item difficulty, $\mathrm{r}=[(\mathrm{M₁}-\mathrm{M₀})/\mathrm{S}]\times&radic;(\mathrm{p}(1&minus;\mathrm{p}))$.

Published studies of AI-generated items have used point-biserial criteria of 0.30 [15,16], while another treated values above 0.20 as acceptable and above 0.30 as preferable [17]. These values illustrate published decision criteria; because the present framework specifies the corrected item-rest coefficient, they should not be transferred mechanically. The programme must prespecify the criterion it will apply to the corrected formulation. An alternative index is $\mathrm{D}=\mathrm{p_upper}-\mathrm{p_lower}$, the difference in proportion correct between the upper and lower 27% of examinees [9].

A non-functioning distractor is an option selected by fewer than 5% of examinees [9]. An item retaining several such options operates, in practice, with fewer functioning options than it declares, and the gate returns it for revision of the options rather than for rejection of the item.

Internal consistency is a property of the form, not of the item, and is reported as Cronbach's α or the Kuder-Richardson Formula 20 (KR-20). The gate requires that the programme prespecify which coefficient it will report, the minimum value coherent with the intended use of the scores, the number of items, and a confidence interval; α is influenced by test length, and the item count should therefore accompany its interpretation.

DIF asks whether examinees of equal ability but different group membership have unequal probability of a correct response. The plan must prespecify the detection method, Mantel-Haenszel, logistic regression, or an item response theory (IRT)-based approach, the subgroup size adequate for that method, the flagging rule, and the substantive review that follows a flag. Sample-size requirements differ appreciably between methods, so method and sample size must be chosen together [10]. A flagged item is not thereby shown to be biased: DIF detection initiates content review, and only that review can establish whether the difference is construct-irrelevant. Where the required subgroup size is not reached, the gate requires that this be recorded, not that the analysis be omitted silently.

Under IRT, the corresponding quantities are model parameters on a latent scale rather than sample proportions, and CTT thresholds do not transfer to them. Difficulty is the location parameter b, expressed in the metric of examinee ability, so the direction of the scale is inverted relative to p: higher b denotes a harder item and higher p an easier one. Discrimination is the slope parameter a in models that estimate it, such as the two- and three-parameter models; under the Rasch or one-parameter model, discrimination is constrained equal across items and is not an item-level parameter available for review. Any programme adopting IRT should state its model, estimation method, and sample-size assumptions in the Gate 6 plan, because parameter stability depends on all three [7].

Reproducibility of gate decisions

Four of the seven gates, clinical review, item-writing quality, blueprint alignment, and final approval, rest on expert judgment. Without demonstrated reproducibility, the workflow records expert judgment but does not establish the consistency of that judgment, and the record it produces will not support the use to which it is later put.

The gate therefore specifies a reproducibility design rather than a statistic. A proportion of artifacts, fixed in advance, is rated independently by two reviewers blinded to each other's decision. Agreement is computed on those independent ratings, before any adjudication; consensus reached by discussion may conceal low initial reproducibility, and a figure computed after adjudication measures the discussion rather than the instrument. Disagreements are then adjudicated, and the adjudication is recorded separately from the agreement statistic.

The statistic follows from the measurement scale and the number of raters rather than from preference. For two reviewers assigning the ordinal outcome categories recorded at final approval, namely, rejected, requires modification, approved for drafting or teaching support only, approved for pilot testing, and approved for scored use, weighted Cohen's kappa is appropriate, and the plan must state whether linear or quadratic weights will be used, since the two penalize distant disagreement differently and are not interchangeable. Where more than two reviewers rate the same artifact, Fleiss' kappa provides a multirater coefficient, but it treats the categories as unordered; a programme wishing to preserve the ordinal structure with more than two raters should state how it will do so rather than adopt Fleiss' kappa by default. Where a gate produces a continuous rating, such as a summed item-writing quality score, an intraclass correlation coefficient is appropriate, and the plan must state the model, whether single or average measures are reported, and whether absolute agreement or consistency is the target. For deciding whether different reviewers reach the same decision, absolute agreement is the pertinent definition; consistency would credit reviewers who rank artifacts alike while applying systematically different thresholds.

Every coefficient is reported with a 95% confidence interval. A kappa reported without an interval conceals how much of its value the sample size can support, and small reliability samples yield intervals compatible with both adequate and inadequate agreement.

The minimum acceptable level of agreement is prespecified according to the consequence of the gate and functions as a stop condition: where it is not reached, the process halts for recalibration of reviewers or revision of the rating instrument before further artifacts are processed. Reproducibility evidence thus enters the workflow on the same terms as the psychometric plan: declared in advance, reviewed against what was declared, and capable of stopping the line.

This technical report describes the workflow rather than an implementation of it and reports no reliability data. That is a limitation of the present work and the first quantity a prospective evaluation should report.

A worked example

The following example is illustrative and hypothetical. It contains no learner data and no measured item statistics; the values mentioned are placeholders used to show where each decision occurs, not results obtained in practice.

A generative model is prompted to produce a single-best-answer MCQ on the initial management of community-acquired pneumonia in an adult outpatient. At Gate 1, the artifact is registered as a complete MCQ intended for scored use, which sets the evidentiary burden at its highest level. At Gate 2, the generating tool, the prompt, and the accountable reviewer are recorded.

At Gate 3, a subject matter expert confirms that the stem is clinically accurate, that the correct answer follows current guidance, and that no unsafe recommendation is present. The item passes.

At Gate 4, it does not. Two of the four options are implausible for any candidate who has reached the level being tested, and the correct answer is noticeably longer than the alternatives: a length cue that introduces construct-irrelevant variance. The artifact is classified as requiring modification and returns to item writing. The distractors are rewritten to represent recognizable clinical errors rather than implausible ones, and the options are balanced in length. On its second passage, the item clears Gate 4.

At Gate 5, the item is mapped to a defined respiratory-infection competency in the blueprint. At Gate 6, on first passage, the review team records that this item, being intended for scored use, requires pilot testing with item difficulty, discrimination, and distractor analysis and that DIF will be examined because scores will be compared across cohorts. No data exist yet; what exists is the plan. At Gate 7, the item is therefore approved for pilot testing, not for scored use.

After the pilot, the item returns to Gate 6. Difficulty and discrimination fall within the range the plan defined as acceptable, but one distractor is selected by almost no examinee and is judged non-functioning. This is not grounds for rejection: the item is again classified as requiring modification, the distractor is replaced, and the revised item re-enters the pilot cycle. Only when the second set of results is reviewed and accepted at Gate 6 does the item reach Gate 7 as approved for scored use.

The path illustrates three properties of the workflow. Clinical accuracy alone did not carry the item through, since it failed structural review after passing expert review. Failure at a gate returned the artifact for revision rather than ending its life. And the psychometric gate was decisive twice: once to require evidence and once to judge it.

## Discussion

This technical report contributes a compact, auditable control workflow for integrating AI-generated assessment artifacts into medical education. By separating artifact taxonomy, expert review, item-writing review, blueprint alignment, psychometric readiness, and final approval, the workflow aims to prevent premature deployment of plausible but unvalidated material. It requires assessment teams to document what has and has not been verified before an artifact is used for teaching support, piloting, or scored assessment. Because the gates are traversed iteratively rather than once, the same documentation also records why an artifact was returned for revision, which preserves the reasoning behind a decision and not only its outcome.

This workflow aligns with recent literature showing both the promise and the limits of AI-generated MCQs. Reviews of AI-generated MCQs in medical education report increasing interest in prompt design, expert ratings, validity evidence, and psychometric outcomes, but they also caution that available evidence remains uneven and does not support unsupervised use for high-consequence assessment [1,4]. Comparative psychometric studies have begun to examine whether AI-generated items resemble clinician-authored items in difficulty, discrimination, and point-biserial performance, but such findings are context-specific and should not be generalized without local evidence [5].

The framework proposed here is complementary to the empirical literature on AI-generated assessment material rather than in competition with it. That literature has largely asked whether items generated by large language models perform acceptably; the present workflow asks what must be established before such an item is used at all.

Kıyak et al. incorporated two ChatGPT-generated case-based MCQs into a rational pharmacotherapy examination taken by 99 fourth-year medical students, after expert panel review and without substantive content modification; patient names were replaced with generic terms for cultural considerations. Both items exceeded a point-biserial criterion of 0.30 (0.41 and 0.39), yet one retained three non-functioning options, defined as options chosen by fewer than 5% of examinees [15]. An item may therefore satisfy a discrimination criterion while failing an option-quality criterion, which is why the workflow specifies item-writing quality and psychometric readiness as separate gates rather than as a single checkpoint.

Coşkun et al. randomized 74 medical students to ChatGPT-generated or human-written ill-defined cases in an evidence-based medicine programme and separately administered 15 ChatGPT-generated MCQs. Student ratings of the cases did not differ significantly between groups, while only six of the 15 items reached a point-biserial correlation of 0.30 [16]. The two findings concern different objects, learner perception of cases and psychometric behavior of items, and the study therefore illustrates why qualitative acceptability and psychometric evidence should not be collapsed into a single gate.

Laupichler et al. compared 25 human-written and 21 ChatGPT-generated MCQs answered by 161 students in a formative neurophysiology test. In that study, item difficulty did not distinguish the two sets, whereas discrimination did: mean 0.36 (SD 0.09) for human-written items against 0.24 (SD 0.14) for large language model-generated ones (P = .001). Students identified the source of a question correctly in 57% of cases [17]. Two consequences bear on the present framework: a review process that inspects apparent difficulty is not thereby inspecting discrimination; and the machine origin of an item was not reliably apparent to those answering it, which is a reason to establish provenance by record, as Gate 2 requires, rather than by inspection. Whether expert reviewers would recognize machine authorship more reliably than students was not examined in that study.

Taken together, these studies show that acceptable performance on one dimension does not assure acceptable performance on another. Item difficulty, discrimination, and distractor functioning should therefore be examined separately before scored use, rather than ordered into a hierarchy of importance: the aggregate evidence on AI-generated items remains heterogeneous and does not support ranking one dimension above the others [4].

A related governance problem has been described outside assessment. Fathi et al., writing on radiological diagnosis, argue that AI output requires interpretable confidence information and clinical validation and describe a division of labor in which the system supplies recommendations while the final decision remains with the radiologist [18]. The analogy to assessment is ours, not theirs. A generated item, like a generated diagnosis, carries no intrinsic indication of the confidence it warrants. The evidence that licenses its use must therefore be specified externally and in advance, and the decision to deploy it must remain with an accountable human.

Distractor analysis addresses whether options function as plausible alternatives and contribute to item quality; non-functioning distractors can weaken the interpretability and efficiency of MCQs [9]. DIF analysis addresses fairness by evaluating whether examinees from different demographic groups have different probabilities of answering an item correctly after controlling for the construct being measured [10]. Therefore, both distractor and DIF analyses represent distinct but equally critical components of a comprehensive psychometric evaluation.

This report describes a process and has several limitations. It is a single-team technical report, developed through iterative practice rather than through a formal consensus methodology, and it does not present outcome analyses, item-performance metrics, learner data, or empirical evidence comparing AI-generated items to human-authored items. A further distinction should be kept in view: the workflow requires that psychometric evaluation be planned and, once data exist, reviewed, but planning and reviewing evidence are not themselves evidence that the workflow works. The report does not claim that the proposed workflow improves student learning, reduces faculty workload, or establishes psychometric equivalence. Establishing any such claim would require evaluations the report does not contain: feasibility in routine use, inter-rater reliability among reviewers applying the gates, the faculty workload the workflow imposes, and its measurable effect on item quality. Until those exist, what is offered here is a governance structure for deciding when stronger empirical evidence is required.

The workflow can be adapted by medical education teams using generative AI for MCQs, distractors, answer explanations, feedback prompts, or item variants. Practical guidance on AI-assisted MCQ development emphasizes careful prompting, expert review, and quality assurance rather than autonomous deployment [6]. Accordingly, human governance complements but does not replace formal measurement evidence. AI may assist with drafting, but human reviewers remain responsible for clinical accuracy, and empirical testing remains necessary when artifacts are used for scored or consequential assessment [2,7,10].

## Conclusions

AI-generated medical assessment artifacts should be governed as candidates requiring staged validation rather than treated as ready-made instruments. A structured workflow that separates clinical review, item-writing quality, blueprint alignment, distractor functioning, fairness review, psychometric readiness, and final human approval, and that allows artifacts to return to an earlier stage when a later one reveals a defect, can help authors and assessment teams preserve accountability. Human oversight is necessary for content accuracy and safety, but empirical evidence remains essential when these artifacts are intended for scored or consequential assessment. The workflow proposed here is a governance recommendation; its effectiveness has not been empirically evaluated, and adopting it does not by itself constitute evidence that assessment quality has improved.
